# Asymmetric Synthesis of *α*-Aminophosphonates Using the Inexpensive Chiral Catalyst 1,1’-Binaphthol Phosphate 

**DOI:** 10.3390/molecules15085782

**Published:** 2010-08-24

**Authors:** Weiming Xu, Sha Zhang, Song Yang, Lin-Hong Jin, Pinaki S. Bhadury, De-Yu Hu, Yuping Zhang

**Affiliations:** State Key Laboratory Breeding Base of Green Pesticide and Agricultural Bioengineering, Key Laboratory of Green Pesticide and Agricultural Bioengineering, Ministry of Education, Guizhou University, Guiyang 550025, China; E-Mails: xuweiming2009@163.com (W.X.); lisarik@126.com (S.Z.); fcc.jinlh@gzu.edu.cn (L.-H.J.); bhadury@gzu.edu.cn (P.-S.B.); fcc.dyhu@gzu.edu.cn (D.-Y.H.); zhangyupinggz@163.com (Y.Z.)

**Keywords:** *α*-aminophosphonate derivatives, aldimines, cinnamaldehyde, chiral organo- catalyst, asymmetric addition

## Abstract

Asymmetric addition under mild conditions of dialkyl phosphites on aldimines derived from cinnamaldehyde catalyzed by the inexpensive chiral organocatalyst (*R*)-3,3’-[4-fluorophenyl]_2_-1,1’-binaphthol phosphate has been found effective to give new α-amino-phosphonates **9** in moderate yields (30–65%) and enantiomeric excess (8.4%–61.9%).

## 1. Introduction

**Scheme 1:**
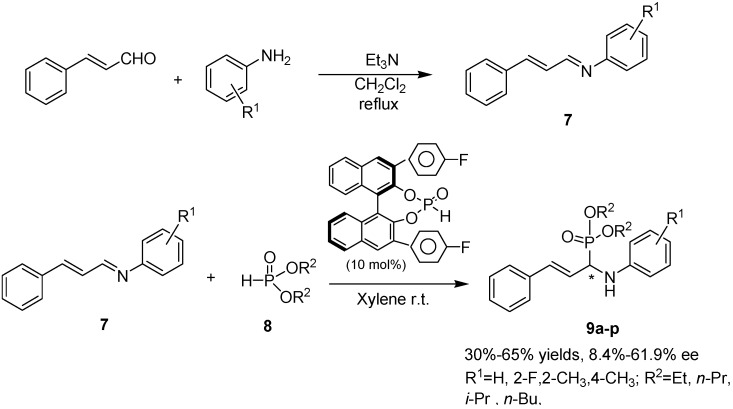
Synthetic route to title chiral compounds **9**.

*α*-Aminophosphonic acids, structural analogues of amino acids, are known for their diversity in agricultural and biological applications. A large number of *α*-aminophosphonic acids and their phosphonate esters and a few short peptides of natural and synthetic origin bearing similar structural features exhibit enzyme inhibitory [1,2,3,4,5,6,7,8,9,10,11], antibiotic [12], antibacterial [13,14,15], antiviral [16], antifungal [17], and herbicidal activities [18]. As the biological activity of *α*-aminophosphonates is markedly influenced by the absolute configuration of the *α*-carbon atom directly linked to the phosphorous center [19], the synthesis of optically pure enantiomers of *α*-aminophosphonates and their derivatives with desired property constitutes an important task in organic synthesis [20]. The strategy for this challenging asymmetric transformation mostly relies upon catalytic hydrophosphonylation of either a preformed or *in situ* generated imine with dialkyl phosphates [21,22,23,24,25,26]. Since these reactions typically involve Mannich type nucleophilic attack of a phosphite at the electrophilic imine, both the components of the reaction can be simultaneously activated, at least in principle, by a bifunctional organocatalyst. Based on this concept, Akiyama *et al.* [27] have designed an enantioselective hydrophosphonylation of preformed aromatic and unsaturated imines catalyzed by the axially chiral binaphthyl phosphoric acid derivative **6b** affording *α*-aminophosphonates with an enantiomeric excess of up to 90%. The same catalyst was subsequently utilized by our group [28] in a highly enantioselective preparation of a series of fluorine containing asymmetric *α*-aminophosphonates through hydrophosphonylation of aldimines mostly derived from cinnamaldehyde. Due to the growing concern for the influence of the nature of the substrate and the catalyst structure on the enantiomerically pure final asymmetric hydrophosphonylation of aldehydes and imines, organic reactions with use of conventional organic chiral catalyst have attracted the attention of synthetic organic chemists. A number of chiral binaphthyl phosphoric acid derivative catalysts such as **6b**, with varying substituents at the 3- and 3′-positions of the binaphthyl scaffold have been extensively studied recently [29,30]. With this information in hand, we proceeded to synthesize enantiopure *α*-aminophosphonates with heterocycle moieties to investigate their biological activities. We found however that the enantioselectivity of chiral Brønsted acid **6b-** catalyzed enantioselective hydrophosphonylations of imines with benzothiazoles moieties was very poor [31]. This indicates that the structures of imine and catalyst play an important role in affecting the reaction’s enantioselectivity. Herein we studied the effect of a relatively simple and inexpensive catalyst (R)-3,3’-[4-fluorophenyl]_2_-1,1’-binaphthol phosphate (**6a**) which was obtained from easily accessible 4-bromofluorobenzene. The bulky 3,3’ aryl substituents of catalyst **6b **has been replaced in this catalyst by a less sterically demanding 4-fluorophenyl group. The synthetic route to the asymmetric *α*-aminophosphonates in presence of chiral catalyst is depicted in Scheme 1. The structures of the target compounds were firmly established by IR, ^1^H-, ^13^C-, ^31^P- and ^19^F-NMR spectra and elemental analysis. 

## 2. Results and Discussion

**Scheme 2:**
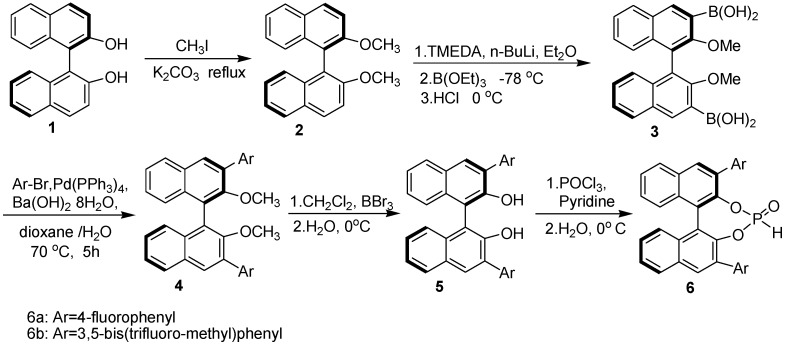
Synthetic route to chiral catalysts **6a** and **6b**.

The catalysts **6a** and **6b** were prepared from starting material *R*-BINOL through a five step synthetic sequence [32] involving etherification, boronation, Suzuki coupling, demethylation, and phosphorylation under a strictly inert atmosphere, as is shown in Scheme 2. 

The suitability of the imine structure for enantioselective catalytic synthesis of chiral *α*-amino-phosphonates was studied first and the results are shown in Table 1. In line with our earlier observation [28] that catalyst **6b** was more suited to cinnamaldehyde-derived imines (entry 4) compared to the one derived from benzaldehyde and heterocyclic amine (entry 2), the catalyst **6a** showed too a similar trend with cinnamaldehyde in enhancing the enantioselectivity (entry 1 *vs*. entry 3).

**Table 1:** Effect of Imine Structure on Enantioselectivity.^ a^ 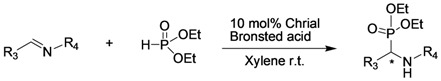

Entry	Catalyst	R_3_	R_4_	Yield (%)	*e*.*e*. (%)^b^
1	6a	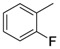	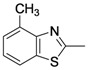	20	0
2	6b	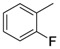	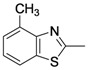	56	10.2
3	6a	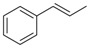	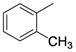	51	31.9
4	6b	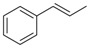	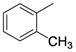	45	89.5

^a ^Reaction conditions: aldimine (1 mmol), catalyst **6a** or **6b **(0.1 mmol), xylene (15 mL), diethyl phosphite (2 mmol), room temp. for 24 h; ^b ^Determined by chiral HPLC.

Having established cinnamaldehyde as the ideal substrate for the reaction, it was reacted with different aromatic amines (Table 2) to generate aldimines for further conversion into *α*-amino-phosphonates. The formation of these imines is generally accompanied by side products due to the possibility of a 1,4-Michael attack on cinnamaldehyde by a nucleophile/base. The product **7** was obtained in relatively low yield in protic solvent, while at elevated reaction temperature Michael adducts started to appear. Under optimized conditions, the new cinnamaldehyde imines were prepared by refluxing the aldehyde and amine components in methylene chloride followed by recrystallization from ethanol. Whilst the low boiling methylene chloride restricts the formation of Michael addition product, the use of inert atmosphere prevents undesired oxidation of aldehyde into the acid. Activation of the amine by a weak organic base e.g. triethylamine and addition of molecular sieves were found advantageous to improve the yield of the imine. The desired aldimines **7** from different amines were obtained in 65–85 % yield, as shown in Table 2*.*

**Table 2:** Synthesis of imine **7**.

Entry	7	R	Time (h)	Yield (%)
1	7a	H	3	85
2	7b	2-F	5	89
3	7c	2-CH_3_	8	77
4	7d	4-CH_3_	8	65

^a ^Reaction conditions: aniline (5 mmol), aldehyde (5 mmol), triethylamine (0.2 mL), CH_2_Cl_2_ (15 mL), 40 ºC; ^b^ Yields refer to isolated pure compounds.

**Figure 1:**
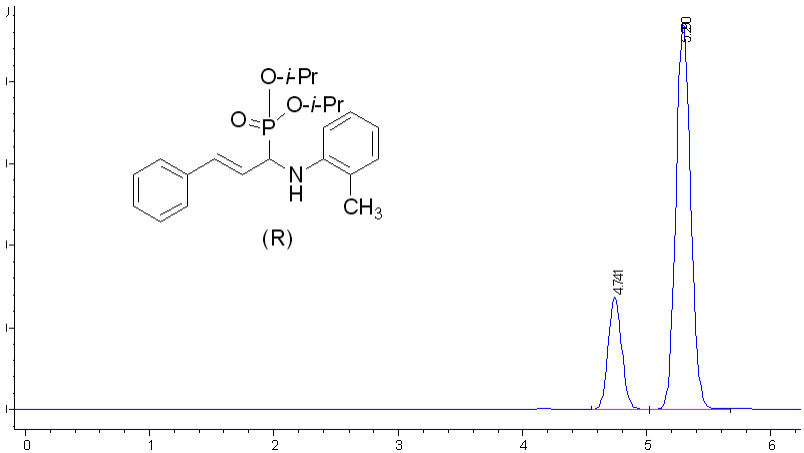
Representative chiral HPLC chromatogram of enantioenriched *α*-aminophosphonate **9k**.

Addition of dialkyl phosphite **8 **via its nucleophilic phosphite tautomer bearing the Bronsted acidic OH group to cinnamaldehyde imine **7** in presence of catalyst **6a** then leads to the generation of title chiral *α*-aminophosphonates **9** as listed in Table 3. The imine and the phosphite are presumed to be simultaneously activated by the phosphoric acid hydrogen and phosphoryl oxygen of the catalyst respectively through a nine membered transition state [28]. The stereochemical course of the attack is controlled by the aryl groups at the 3,3’-positions of the catalyst. The scope of various dialkyl phosphites in the asymmetric hydrophosphonylation reaction was investigated first. As evident from the data presented in Table 3, similar to the observation made by Akiyama *et al*. [27], diisopropyl phosphite afforded the highest enantioselectivity (61.9%). This is expected because as per the proposed model, the OH moiety of dialkyl phosphite should play a significant role in conferring enantioselectivity. Irrespective of the nature of amine, the trend in enantioselectivity generally followed the order *i*-Pr > *n*-Bu > Et > *n*-Pr. As for different aromatic amines, electron donating substituent such as methyl group on the aryl ring seems to have a positive influence on ee of the target compounds to a certain degree. We reasoned that the hydrogen bond activation of imine induced by phosphoric acid catalyst should be more favorable in presence of electron donating groups. It was also found that imine obtained from o-methyl aniline displayed greater enantioselectivity compared to the one derived from p-methyl aniline. The results shown in Table 3 afforded only moderate yield, and the outcome depended on, to some extent, the substrate and the nucleophilic phosphite structures. A representative chiral HPLC chromatogram of **9k **is shown in Figure 1. 

**Table 3:** Organocatalytic enantioselective reaction with chiral phosphoric acid **6a**.

Entry	Compound	R_1_	R_2_	Yield (%)	*e.e.* (%)
1	**9a**	H	Et	41	16.5
2	**9b**	H	Pr	52	17.3
3	**9c**	H	*i*-Pr	33	36.9
4	**9d**	H	Bu	65	16.7
5	**9e**	2-F	Et	60	18.8
6	**9f**	2-F	Pr	54	8.4
7	**9g**	2-F	*i*-Pr	31	44.2
8	**9h**	2-F	Bu	47	27.5
9	**9i**	2-CH_3_	Et	51	31.9
10	**9j**	2-CH_3_	Pr	48	16.9
11	**9k**	2-CH_3_	*i*-Pr	32	61.9
12	**9l**	2-CH_3_	Bu	48	30.1
13	**9m**	4- CH_3_	Et	50	28.3
14	**9n**	4- CH_3_	Pr	45	15.3
15	**9o**	4- CH_3_	*i*-Pr	30	51.5
16	**9p**	4- CH_3_	Bu	39	22.7

^a ^Reaction conditions:. aldimine (10 mmol), catalyst **6a** (56 mg, 0.1 mmol), xylene (15 mL), dialkyl phosphite **8 **(1 mmol), room.temp; ^b ^Determined by chiral HPLC; ^c ^Yields refer to isolated pure compounds.

In order to optimize the reaction parameters for the preparation of **9k**, its enantioselective synthesis was carried out under different conditions, as shown in Table 4. Amongst the various solvents selected for the study (acetonitrile, methylene chloride, toluene and xylene, entries 1–4), xylene afforded the highest enantioselectivity under identical condition of temperature and catalyst loading. When the reaction was executed at room temperature with reduced catalyst amount (entries 6 and 7), enantioselectivity dropped significantly whereas on enhancing the loading (entry 5), no significant improvement was noticed compared to 10% loading (61.9% ee, entry 4). As expected for this type of catalytic reaction, enantioselectivity was lowered when the temperature was raised to 50°C (entry 10) and at lower temperatures (entries 8 and 9) ee was enhanced, but only marginally. Therefore, the enantioselective synthesis of **9k** was best conducted in xylene at room temperature with 10 mol% loading of catalyst **6a** (entry 4).

Further, based on the sign of optical rotation of **9k**, its absolute configuration in the presence of catalyst (*R*)-**6a** was proposed as (*R*) by comparison with literature data [20,28]. The proposed stereochemical model of the transition state was discussed in earlier work [23,27,28]. 

**Table 4:** Optimization of reaction parameters for enantioselective synthesis of **9k** using chiral catalyst **6a**.

Entry	Solvent	Temp (°C)	Cat 6a (mol%)	ee (%)
1	acetonitrile	r.t	10	30.1
2	methylene chloride	r.t	10	48.0
3	toluene	r.t	10	52.8
4	xylene	r.t	10	61.9
5	xylene	r.t	20	65.1
6	xylene	r.t	5	49.9
7	xylene	r.t	2	20.9
8	xylene	0	10	64.9
9	xylene	-40	10	68.3
10	xylene	50	10	57.9

## 3. Experimental

### 3.1. General

The melting points of the products were determined on a XT-4 binocular microscope (Beijing Tech Instrument Co., China) and were not corrected. The IR spectra were recorded on a Bruker VECTOR22 spectrometer in KBr disks. Elemental analysis was performed on an Elementar Vario-III CHN analyzer. The reagents were all of analytical grade or chemically pure. Analytical TLC was performed on silica gel GF254. ^1^H-NMR and ^13^C-NMR spectra were recorded on a JEOL-ECX 500 instrument (500 MHz for ^1^H, 125 MHz for ^13^C, 200 MHz for ^31^P, 470 MHz for ^19^F) using CDCl_3_, acetone *d_6_*, DMSO *d_6_* as solvent. TMS (*δ* = 0) served as an internal standard for ^1^H-NMR, CDCl_3_ (*δ* = 77.0) was used as an internal standard for ^13^C-NMR, H_3_PO_4_ (*δ* = 0) was used as an internal standard for ^31^P-NMR, CF_3_COOD was used as an external standard (*δ* = −76.5) for ^19^F-NMR. IR spectra were recorded on a Bruker VECTOR32 spectrometer and are reported in terms of frequency of absorption. Elemental analysis was performed on an Elementar Vario-III CHN analyzer. The HPLC was carried out on an Agilent 1100 series apparatus equipped with DAD detector using Chiralpak AD-H-amylose tri(3,5-dimethylphenylcarbamate) coated on 5 μm silica-gel and Chiralpak IA-amylose tri(3,5-dimethylphenylcarbamate) immobilized on silica-gel columns (each of 250 × 4.6 mm i.d., Daicel Chemical Industries Ltd). All solvents were purified and dried according to standard procedure. Organic solutions were concentrated under reduced pressure on a Buchi rotary evaporator. Molecular sieves (5Ǻ) were flame activated and the reactions were monitored by TLC with Huanghai GF254 silica-gel coated plates. The intermediates **6a-b and 7 **were prepared according to the literature methods as described [25,27]. 

### 3.2. Preparation of chiral catalysts ***6a-b***

*(R)-3,3’-[4-fluorophenyl]_2_-1,1’-binaphthyl phosphate* (**6a**): This compound was obtained as a white solid, m.p. > 290 °C; [α]_D_^20 ^= -291.2 (c = 0.04, CHCl_3_); IR (KBr cm^-1^): *ν* 3419 (OH), 2968 (C-H), 1382 (C-F), 1249 (P=O). ^1^H-NMR (DMSO-*d*_6_,): *δ* 8.11(q, *J =* 4.70 Hz, 2H, Ar-H ), 8.03–8.06(m, 2H, Ar-H), 7.41(t, *J =* 7.20 Hz, 1H, Ar-H), 7.24–7.31(m, 3H, Ar-H), 7.02 (d, *J =* 8.60 Hz, 1H, Ar-H);^ 13^C-NMR (CDCl_3_): *δ* 163.2, 161.2, 148.0, 134.9, 133.8, 132.9, 132.3, 130.5, 128.9, 126.4, 125.2, 123.1, 115.0; ^31^P-NMR (CDCl_3_): *δ* 4.64; ^19^F-NMR (CDCl_3_) : *δ* -115.6.

*(R)-3,3’-[3,5-bis(trifluoromethyl)phenyl]_2_-1,1’-binaphthyl phosphate* (**6b**): This compound was obtained as a white solid, m.p > 250 °C, [α]_D_^20 ^= -195.1 (c= 0.9, CHCl_3_). ^1^H-NMR (DMSO-*d*_6_,): *δ* 8.24 (s, 4H), 7.94–7.98 (m, 4H), 7.81 (s, 2H), 7.47 (t, *J* = 7.15 H_Z_, 2H), 7.26–7.33 (m, 4H); ^13^C-NMR (CDCl_3_): *δ* 145.5, 140.2, 132.8, 131.5, 131.2, 130.9, 130.7, 130.3, 128.5, 127.2, 126.9, 126.7, 125.9, 124.5, 123.3, 122.4, 120.8, 120.2; ^31^P-NMR (CDCl_3_): *δ* 5.77; ^19^F-NMR (CDCl_3_): *δ* ‑62.7. 

### 3.3. Preparation of intermediate imines ***7***

To a 50 mL three necked round-bottomed flask equipped with a stirring bar, were placed cinnamaldehyde (5 mmol), aniline (5 mmol ) and methylene chloride (15 mL). Once a clear solution was obtained, freshly distilled dry triethylamine (2 mL) and molecular sieves (5Å) were added. The reaction mixture was first stirred for 5 min at room temperature and then refluxed for the indicated time shown in Table 2. The reaction mixture was filtered and the solvent was removed under reduced pressure. The crude product obtained was recrystallized from ethanol to afford a yellow solid.

*3-Phenylallylideneaminobenzene* (**7a**): Yield 85%, m.p. 105–107 °C; IR (KBr cm^-1^): *ν* 1620 (C=N); ^1^H-NMR (CDCl_3_): *δ* 8.26 (d, *J* = 8.00 Hz, 1H, Ar-H), 7.53 (d, *J =* 6.85 Hz, 2H, Ar-H), 7.34–7.41(m, 5H, Ar-H), 7.09–7.25(m, 5H, Ar-H + -CH=); ^13^C-NMR (CDCl_3_): *δ* 161.7, 151.8, 144.1, 135.6, 129.7, 129.2, 129.0, 128.6, 127.6, 126.2, 121.0.

*2-(3-Phenyl-allylideneamino)fluorobenzene* (**7b**): Yield 89%, m.p. 72–73 °C; IR (KBr cm^-1^): *ν* 1629 (C=N); ^1^H-NMR (CDCl_3_): *δ* 8.13–8.20 (m, 1H, Ar-H), 7.54 (d, *J =* 6.90 Hz, 2H, Ar-H), 7.34–7.41 (m, 3H, Ar-H), 7.10–7.21(m, 4H, Ar-H + -CH=), 6.86(d, *J =* 7.45 Hz, 1H, -CH=), 2.30(s, 3H, CH_3_);^ 13^C-NMR (CDCl_3_): *δ* 161.5, 151.3, 143.6, 135.7, 131.7, 130.3, 129.6, 129.0, 128.8, 127.5, 126.7, 125.7, 117.8, 17.9.

*2-(3-Phenylallylideneamino)-methylbenzene* (**7c**): Yield 77%, m.p. 71–73 °C; IR (KBr cm^-1^): *ν* 1627 (C=N); ^1^H-NMR (CDCl_3_): *δ* 8.13–8.20 (m, 1H, Ar-H), 7.54 (d, *J =* 6.90 Hz, 2H, Ar-H), 7.34-7.41 (m, 3H, Ar-H), 7.10–7.21 (m, 4H, Ar-H + -CH=), 6.86 (d, *J =* 7.45 Hz, 1H, -CH=), 2.30 (s, 3H, CH_3_); ^13^C-NMR (CDCl_3_): *δ* 161.5, 151.3, 143.6, 135.7, 131.7, 130.3, 129.6, 129.0, 128.8, 127.5, 126.7, 125.7, 117.8, 17.9.

*4-(3-Phenylallylideneamino)-methylbenzene* (**7d**): Yield 65%; m.p. 76–77 °C; IR (KBr cm^-1^): *ν* 1625 (C=N); ^1^H-NMR (CDCl_3_): *δ* 8.27–8.29 (m, 1H, Ar-H), 7.52–7.54 (m, 2H, Ar-H), 7.33–7.40 (m, 3H, Ar-H), 7.08–7.19 (m, 6H, Ar-H + -CH=), 2.36 (s, 3H, CH_3_); ^13^C-NMR (CDCl_3_): *δ* 160.8, 149.1, 143.6, 136.1, 135.7, 129.9, 129.5, 129.0, 128.8, 127.5, 120.9, 21.1.

### 3.4. Preparation of title chiral compounds ***9a-p***

The mixture of aldimine (1 mmol) and the catalyst **6a** (0.1 mmol, 0.056 g) in xylene (15 mL) was stirred for 15 min at room temperature. When a yellow suspension resulted, dialkyl phosphite was added and the reaction mixture was further stirred at room temperature for 24 h. The reaction was stopped by the addition of aqueous NaHCO_3_ and the solution was extracted with ethyl acetate. The combined organic layer was washed with brine, dried over anhydrous Na_2_SO_4_, and concentrated under reduced pressure. The residue was purified by preparative chromatography (eluent: petroleum ether/ ethyl acetate = 3/1) to provide the product in 30~65% yield, which was further subjected to chiral HPLC analysis on a Daicel Chiralpak IA column. Considering the effect of enatiomer self-disproportionation [33], we loaded a smple of compound **9k **of 61.9% ee on a regular gel column and subjected the sample to chromatography with petroleum ether-ethyl acetate = 3/1, *v*/*v*, then the ee value of sample 9k purified by preparative chromatography was determined on a chiral stationary phase (IA column), which was as the same as the original compound **9k**. Also, using literature method [34] to make it, no sublimation was found for the title compound **9k** over the temperature range from R.T to 50 °C.

*Diethyl 1-[N-(phenyl)amino]-3-phenyl-2-propenylphosphonate* (**9a**): White solid, yield 41%; m.p. 80.0–81.5 °C; IR (KBr cm^-1^): *ν* 3292 (N-H), 2985 (C-H), 1600 (C=C), 1496 (C=C), 1222 (P=O), 1020 (P-O-C); ^ 1^H-NMR (CDCl_3_): *δ* 7.16–7.36(m, 6H, Ar-H), 6.68–6.76 (m, 4H, Ar-H), 6.24 (tt, *J =* 5.72, 5.42 Hz, 1H, CH-P), 4.43 (tt, *J =* 6.85, 6.87 Hz, 1H, -CH=), 4.28 (t, *J =* 8.02 Hz, 1H, -CH=), 4.15–4.20 (m, 4H, CH_2_O), 1.29 (t, *J =* 7.4Hz, 6H, CH_3_); ^13^C-NMR (CDCl_3_): *δ* 146.6, 136.2, 132.9, 129.3, 128.6, 127.9, 126.6, 123.5,118.6, 113.8, 63.5, 54.7, 53.8, 16.6; ^31^P-NMR (CDCl_3_): *δ* 22.3; Anal. Calcd for C_19_H_24_NO_3_P: C 66.07, H 7.00, N 4.06; Found: C 65.99, H 7.18, N 4.19; Analysis: Daicel Chiralpak IA (hexane-EtOH = 90:10 / *v*/*v*), Flow rate = 1.0 mL/min, UV = 254 nm, t_R _(minor) = 8.45 min (*R*), t_R _(major) = 10.25 min (*S*). *e.e.* 16.5%.

*Di-n-propyl 1-[N-(phenyl)amino]-3-phenyl-2-propenylphosphonate* (**9b**): White solid, yield 52%; m.p. 59.6–61.1 °C; IR (KBr cm^-1^): *ν* 3317 (N-H), 2966 (C-H), 1602 (C=C), 1498 (C=C), 1230 (P=O), 1015 (P-O-C); ^ 1^H-NMR (CDCl_3_): *δ* 7.15–7.36(m, 6H, Ar-H), 6.68–6.76(m, 4H, Ar-H), 6.24(tt, *J =* 5.45, 5.42 Hz, 1H, CH-P), 4.40 (br, 1H, -CH=), 4.30 (br, 1H, -CH=), 4.02–4.09 (m, 4H, CH_2_O), 1.65 (t, *J =* 6.85 Hz, 4H, CH_2_), 0.91 (t, *J =* 7.45 Hz, 6H, CH_3_); ^13^C-NMR (CDCl_3_): *δ* 146.5, 136.3, 132.9, 129.3, 128.6, 127.9, 126.6, 126.6, 118.6, 113.8, 68.4, 54.7, 53.5, 23.9, 10.1; ^31^P-NMR (CDCl_3_): *δ* 22.8; Anal. Calcd for C_21_H_28_NO_3_P: C 67.54, H 7.56, N 3.75; Found: C 67.31, H 7.56, N 3.63; Analysis: Daicel Chiralpak IA (hexane-EtOH = 90:10 / *v*/*v*), Flow rate = 1.0 mL/min, UV = 250 nm, t_R _(minor) = 7.38 min (*R*), t_R _(major) = 8.31 min (*S*). *e.e.* 17.3%.

*Diisopropyl 1-[N-(phenyl)amino]-3-phenyl-2-propenylphosphonate* (**9c**): White solid, yield 33%; m.p. 81.4–82.5 °C; IR (KBr cm^-1^): *ν* 3294 (N-H), 2976 (C-H), 1600 (C=C), 1498 (C=C), 1224 (P=O), 981 (P-O-C);^ 1^H-NMR (CDCl_3_): *δ* 7.15–7.35 (m, 4H, Ar-H), 6.67–6.75 (m, 4H, Ar-H), 6.24 (tt, *J =* 5.70, 5.45 Hz, 1H, CH-P), 4.74–4.77 (m, 2H, CH-O), 4.37 (br, 1H, -CH=), 4.26 (br, 1H, -CH=), 1.33–1.35 (m, 6H, CH_3_); ^13^C-NMR (CDCl_3_): *δ* 146.7, 136.5, 132.3, 129.3, 128.6, 127.7, 126.6, 123.9, 118.4, 113.8, 71.7, 55.1, 53.9, 24.0; ^31^P-NMR (CDCl_3_): *δ* 21.0; Anal. Calcd for C_21_H_28_NO_3_P: C 67.54, H 7.56, N 3.75; Found: C 67.79, H 7.93, N 3.79; Analysis: Daicel Chiralpak IA (hexane-EtOH = 90:10 / *v*/*v*), Flow rate = 1.0 mL/min, UV = 254 nm, t_R _(minor) = 6.56 min (*R*), t_R _(major) = 7.26 min (*S*). *e.e.* 36.9%.

*Di-n-butyl 1-[N-(phenyl)amino]-3-phenyl-2-propenylphosphonate* (**9d**)**:** White solid, yield 65%; m.p. 53.1–54.7 °C; IR (KBr cm^-1^): *ν* 3290 (N-H), 2956 (C-H), 1602 (C=C), 1498 (C=C), 1219 (P=O), 1024 (P-O-C); ^1^H-NMR (CDCl_3_): *δ* 7.15–7.35 (m, 6H, Ar-H), 6.68–6.76 (m, 4H, Ar-H), 6.24 (tt, *J =* 5.45, 5.45 Hz, 1H, CH-P), 4.43 (tt, *J =* 7.15, 7.15 Hz, 1H, -CH=), 4.27 (t, *J =* 8.30 Hz, 1H, -CH=), 4.07–4.13 (m, 4H, CH_2_O),1.61–1.62 (m, 4H, CH_2_), 1.34 (t, *J =* 7.45 Hz, 4H, CH_2_), 0.87 (d, *J =* 7.45 Hz, 6H, CH_3_);^ 13^C-NMR (CDCl_3_): *δ* 146.5, 136.4, 132.9, 129.3, 128.6, 127.8, 126.6, 123.6, 118.5, 113.8, 66.7, 54.7, 53.5, 32.6, 18.7, 13.6; ^31^P-NMR (CDCl_3_): *δ* 22.9; Anal. Calcd for C_23_H_32_NO_2_P: C 71.66, H 8.37, N 3.63; Found: C 71.51, H 8.47, N 3.55; Analysis: Daicel Chiralpak IA (hexane-EtOH = 90:10 / *v*/*v*), Flow rate = 1.0 mL/min, UV = 254 nm, t_R _(minor) = 7.01 min (*R*), t_R _(major) = 7.72 min (*S*). *e.e.* 16.7%.

*Diethyl 1-[N-(2-fluorophenyl)amino]-3-phenyl-2-propenylphosphonate* (**9e**): White solid, yield 60%; m.p. 60.6–60.9 °C; IR (KBr cm^-1^): *ν* 3280 (N-H), 2985 (C-H), 1616 (C=C), 1525 (C=C), 1325 (C-F), 1236 (P=O), 1018 (P-O-C) ; ^1^H-NMR (CDCl_3_): *δ* 7.21–7.37 (m, 4H, Ar-H), 6.94–7.02 (m, 2H, Ar-H), 6.65–6.73 (m, 3H, Ar-H), 6.26 (tt, *J =* 5.42, 5.45 Hz, 1H, CH-P), 4.16–4.24 (m, 2H, -CH=), 4.12–4.14 (m, 4H, CH_2_O), 1.30 (t, *J =* 8.05 Hz, 6H, CH_3_);^ 13^C-NMR (CDCl_3_): *δ* 152.9, 151.0, 136.2, 135.0, 132.9, 128.6, 128.0, 126.7, 124.6, 123.1, 118.1, 114.7, 113.5, 63.1, 54.5, 53.3, 16.5; ^31^P-NMR (CDCl_3_): *δ* 22.2; Anal. Calcd for C_19_H_23_FNO_3_P: C 62.80, H 6.38, N 3.85; Found: C 62.74, H 6.44, N 3.67; Analysis: Daicel Chiralpak IA (hexane-EtOH = 95:5 / *v*/*v*), Flow rate = 1.0 mL/min, UV = 250 nm, t_R _(minor) = 6.99 min (*R*), t_R _(major) = 9.01 min (*S*). *e.e.* 18.8%.

*Di-n-propyl 1-[N-(2-fluorophenyl)amino]-3-phenyl-2-propenylphosphonate* (**9f**): Yellow oil; yield 54%; IR (KBr cm^-1^): *ν* 3421 (N-H), 2968 (C-H), 1618 (C=C), 1510 (C=C), 1332 (C-F), 1246 (P=O), 997 (P-O-C); ^1^H-NMR (CDCl_3_): *δ* 7.21–7.37 (m, 4H, Ar-H), 6.93–7.02 (m, 2H, Ar-H), 6.67–6.73 (m, 3H, Ar-H), 6.27–6.32 (m, 1H, CH-P), 4.50 (br, 2H, -CH=), 4.02–4.09 (m, 4H, CH_2_O), 1.63–1.70 (m, 4H, CH_2_), 0.90 (t, *J =* 14.9 Hz, 6H, CH_3_);^ 13^C-NMR (CDCl_3_): *δ* 152.9, 151.0, 136.2, 135.0, 132.9, 128.6, 128.0, 126.6, 124.6, 123.2, 118.1, 114.7, 113.4, 68.5, 54.5, 53.3, 24.0, 10.0; ^31^P-NMR (CDCl_3_): *δ* 22.1; Anal. Calcd for C_21_H_27_FNO_3_P: C 64.45, H 6.90, N 3.58; Found: C 64.50, H 6.79, N 3.40; Analysis: Daicel Chiralpak IA (hexane-EtOH = 95:5 / *v*/*v*), Flow rate = 1.0 mL/min, UV = 250 nm, t_R _(minor) = 6.38 min (*R*), t_R _(major) = 7.88 min (*S*). *e.e.* 8.4%.

*Diisopropyl 1-[N-(2-fluorophenyl)amino]-3-phenyl-2-propenylphosphonate* (**9g**): Yellow oil; yield 31%; IR (KBr cm^-1^): *ν* 3421(N-H), 2980(C-H), 1618 (C=C), 1514 (C=C), 1386 (C-F), 1246 (P=O), 989 (P-O-C); ^1^H-NMR (CDCl_3_): *δ* 7.21–7.36 (m, 4H, Ar-H), 6.94–7.02 (m, 2H, Ar-H), 6.64–6.72 (m, 3H, Ar-H), 6.26 (tt, *J =* 5.42, 5.45 Hz, 1H, CH-P), 4.73–4.81 (m, 2H, CH-O), 4.37 (br, 1H, -CH=), 1.33–1.39 (m, 6H, CH_3_), 1.25–1.28 (m, 6H, CH_3_);^ 13^C-NMR (CDCl_3_): *δ* 152.8, 150.9, 136.3, 135.2, 128.6, 127.9, 126.6, 124.6, 123.4, 117.9, 114.7, 113.4, 71.8, 54.9, 53.7, 24.2; ^31^P- NMR (CDCl_3_): *δ* 20.3; Anal. Calcd for C_21_H_27_FNO_3_P: C 64.45, H 6.90, N 3.58; Found: C 64.34, H 6.84, N 3.60; Analysis: Daicel Chiralpak IA (hexane-EtOH = 95:5 / *v*/*v*), Flow rate = 1.0 mL/min, UV = 250 nm, t_R _(minor) = 5.66 min (*R*), t_R _(major) = 7.12 min (*S*). *e.e.* 44.2%.

*Di-n-butyl 1-[N-(2-fluorophenyl)amino]-3-phenyl-2-propenylphosphonate* (**9h**): Yellow oil; yield 47%; IR (KBr cm^-1^): *ν* 3419 (N-H), 2960 (C-H), 1618 (C=C), 1514 (C=C), 1332 (C-F), 1247 (P=O), 1022 (P-O-C); ^1^H-NMR (CDCl_3_): *δ* 7.21–7.36 (m, 4H, Ar-H), 6.93–7.01 (m, 2H, Ar-H), 6.65–6.73 (m, 3H, Ar-H), 6.27–6.31 (m, 1H, CH-P), 4.44 (br, 2H, -CH=), 4.07–4.15 (m, 4H, CH_2_O), 1.60–1.62 (m, 4H, CH_2_), 1.35–1.36 (m, 4H, CH_2_), 0.87–0.88 (m, 6H, CH_3_);^ 13^C-NMR (CDCl_3_): *δ* 152.9, 151.0, 136.2, 135.0, 132.9, 128.6, 128.0, 126.6, 124.6, 123.2, 118.1, 114.7, 113.4, 66.7, 54.5, 53.3, 32.6, 18.7, 13.6; ^31^P-NMR (CDCl_3_): *δ* 22.1; Calcd for C_23_H_31_FNO_3_P: C 65.87, H 7.40, N 3.34; Found: C 65.77, H 7.40, N 3.47; Analysis: Daicel Chiralpak IA (hexane-EtOH = 95:5 / *v*/*v*), Flow rate = 1.0 mL/min, UV = 250 nm, t_R _(minor) = 6.20 min (*R*), t_R _(major) = 7.45 min (*S*). *e.e.* 27.5%.

*Diethyl 1-[N-(2-methylphenyl)amino]-3-phenyl-2-propenylphosphonate* (**9i**): White solid, yield 51%; m.p. 58.5–59.4 °C; IR (KBr cm^-1^): *ν* 3334 (N-H), 2978 (C-H), 1653(C=C), 1521 (C=C), 1230 (P=O), 1018 (P-O-C); ^1^H-NMR (CDCl_3_): *δ* 7.05–7.37 (m, 6H, Ar-H), 6.62–6.71 (m, 3H, Ar-H), 6.27 (tt, *J =* 5.61, 5.72 Hz, 1H, CH-P), 4.52 (br, 1H, -CH=), 4.47 (br, 1H, -CH=), 4.14–4.17 (m, 4H, CH_2_O), 2.25 (s, 3H, CH_3_);^ 13^C-NMR (CDCl_3_): *δ* 144.6, 136.3, 132.8, 130.3, 128.6, 127.9, 127.1, 126.7, 123.8, 122.9, 118.2, 111.2, 63.0, 54.8, 53.6, 17.6, 16.5; ^31^P-NMR (CDCl_3_): *δ* 23.0; Anal. Calcd for C_20_H_26_NO_3_P: C 66.84, H 7.29, N 3.90; Found: C 67.04, H 7.45, N 3.74; Analysis: Daicel Chiralpak IA (hexane-EtOH = 90:10 / *v*/*v*), Flow rate = 1.0 mL/min, UV = 250 nm, t_R _(minor) = 5.95 min (*R*), t_R _(major) = 7.29 min (*S*). *e.e.* 31.9%.

*Dipropyl 1-[N-(2-methylphenyl)amino]-3-phenyl-2-propenylphosphonate* (**9j**): White solid, yield 48%; m.p. 60.0–62.4 °C; IR (KBr cm^-1^): *ν* 3442 (N-H), 2966 (C-H), 1604 (C=C), 1512 (C=C), 1240 (P=O), 999 (P-O-C); ^1^H-NMR (CDCl_3_): *δ* 7.05–7.36 (m, 6H, Ar-H), 6.62–6.70 (m, 3H, Ar-H), 6.26–6.32 (tt, *J =* 5.45, 5.42 Hz, 1H, CH-P), 4.54 (br, 1H, -CH=), 4.02–4.11 (m, 4H, CH_2_O), 2.25(s, 3H, CH_3_), 1.66–1.68 (m, 4H, CH_2_), 0.90–0.93 (m, 6H, CH_3_);^ 13^C-NMR (CDCl_3_): *δ* 144.3, 136.2, 132.8, 130.3, 128.6, 127.8, 127.1, 126.6, 123.9, 122.9, 118.2, 111.2, 68.4, 54.8, 53.5, 24.0, 17.6, 10.1; ^31^P-NMR (CDCl_3_): *δ* 22.9; Anal. Calcd for C_22_H_30_NO_3_P: C 68.20, H 7.80, N 3.62; Found: C 68.11, H 8.05, N 3.40; Analysis: Daicel Chiralpak IA (hexane-EtOH = 90:10 / *v*/*v*), Flow rate = 1.0 mL/min, UV = 250 nm, t_R _(minor) = 6.35 min (*R*), t_R _(major) = 6.33 min (*S*). *e.e.* 16.9%.

*Diisopropyl 1-[N-(2-methylphenyl)amino]-3-phenyl-2-propenylphosphonate* (**9k**): White solid, yield 32%; m.p. 61.8–63.1 °C; [α]_D_^20^ -59.1(c 0.9, CHCl_3_); IR (KBr cm^-1^): *ν* 3387 (N-H), 2974 (C-H), 1604 (C=C), 1510 (C=C), 1230(P=O), 1020 (P-O-C); ^1^H-NMR (CDCl_3_): *δ* 7.04–7.36 (m, 6H, Ar-H), 6.60–6.69 (m, 3H, Ar-H), 6.27 (tt, *J =* 5.42, 5.45 Hz, 1H, CH-P), 4.73–4.78 (m, 2H, CH-O), 4.40 (br, 1H, -CH=), 4.17 (br, 1H, -CH=), 2.25 (s, 3H, CH_3_), 1.33–1.35 (m, 3H, CH_3_), 1.25–1.27 (m, 3H, CH_3_);^ 13^C-NMR (CDCl_3_): *δ* 144.8, 136.5, 132.4, 130.3, 128.6, 127.7, 127.2, 126.6, 124.2, 122.7, 118.0, 111.1, 72.1, 55.2, 54.0, 23.9, 17.6; ^31^P-NMR (CDCl_3_): *δ* 21.1; Anal. Calcd for C_22_H_30_NO_3_P: C 68.20, H 7.80, N 3.62; Found: C 68.50, H 7.75, N 3.51; Analysis: Daicel Chiralpak IA (hexane-EtOH = 90:10 / *v*/*v*), Flow rate = 1.0 mL/min, UV = 250 nm, t_R _(minor) = 4.74 min (*R*), t_R _(major) = 5.29 min (*S*). *e.e.* 61.9%.

*Dibutyl 1-[N-(2-methylphenyl)amino]-3-phenyl-2-propenylphosphonate* (**9l**): White solid, yield 48%; m.p. 62.2–64.1 °C; IR (KBr cm^-1^): *ν* 3400 (N-H), 2958 (C-H), 1604 (C=C), 1510 (C=C), 1244 (P=O), 1022 (P-O-C) ; ^1^H-NMR (CDCl_3_): *δ* 7.05–7.36 (m, 6H, Ar-H), 6.61–6.70 (m, 3H, Ar-H), 6.26 (tt, *J =* 5.45, 5.70 Hz, 1H, CH-P), 4.53 (br, 1H, -CH=), 4.03–4.13 (m, 5H, -CH= + CH_2_O), 2.24 (s, 3H, CH_3_), 1.59–1.64 (m, 4H, CH_2_), 1.32-1.39 (m, 4H, CH_2_), 0.86–0.91 (m, 6H, CH_3_);^ 13^C-NMR (CDCl_3_): *δ* 144.7, 136.4, 132.7, 130.3, 128.6, 127.8, 127.1, 126.6, 123.9, 122.9, 118.2, 111.2, 66.7, 54.7, 53.5, 32.6, 18.7, 17.6, 13.6; ^31^P-NMR (CDCl_3_): *δ* 22.9; Anal. Calcd for C_24_H_34_NO_3_P: C 69.37, H 8.25, N 3.37; Found: C 69.16, H 8.04, N 3.03; Analysis: Daicel Chiralpak IA (hexane-EtOH = 90:10 / *v*/*v*), Flow rate = 1.0 mL/min, UV = 250 nm, t_R_(minor) = 5.17 min (*R*), t_R _(major) = 5.94 min (*S*). *e.e.* 30.1%.

*Diethyl 1-[N-(4-methylphenyl)amino]-3-phenyl-2-propenylphosphonate* (**9m**): Yellow oil; yield 51%; IR (KBr cm^-1^): *ν* 3304 (N-H), 2981 (C-H), 1614 (C=C), 1517 (C=C), 1236 (P=O), 1024 (P-O-C);^ 1^H- NMR (CDCl_3_): *δ* 7.19–7.33 (m, 5H, Ar-H), 6.96 (d, *J =* 9.20 Hz, 2H, Ar-H), 6.67 (dd, *J =* 5.20, 5.20 Hz, 1H, -CH=), 6.61 (d, *J =* 8.60 Hz, 2H, Ar-H), 6.24 (tt, *J =* 5.70, 5.40 Hz, 1H, CH-P), 4.42 (dd, *J =* 6.31, 5.70 Hz, 1H, -CH=), 4.03–4.08 (m, 4H, CH_2_O), 2.21 (s, 3H, CH_3_),1.65-1.68 (m, 6H, CH_3_);^ 13^C- NMR (CDCl_3_): *δ* 144.2, 136.4, 132.9, 129.7, 128.6, 127.8, 126.6, 123.7, 114.0, 63.1, 55.0, 53.8, 20.4, 16.5; ^31^P-NMR (CDCl_3_): *δ* 23.1; Anal. Calcd for C_20_H_26_NO_3_P: C 66.84, H 7.29, N 3.90; Found: C 66.69, H 7.15, N 3.80; Analysis: Daicel Chiralpak IA (hexane-EtOH = 90:10 / *v*/*v*), Flow rate = 1.0 mL/min, UV = 254 nm, t_R _(minor) = 8.08 min (*R*), t_R _(major) = 8.79 min (*S*). *e.e.* 28.3%.

*Dipropyl1-[N-(4-methylphenyl)amino]-3-phenyl-2-propenylphosphonate* (**9n**): Yellow oil; yield 45%; IR (KBr cm^-1^): *ν* 3304 (N-H), 2968 (C-H), 1616(C=C), 1519 (C=C), 1236 (P=O), 1001 (P-O-C);^ 1^H-NMR (CDCl_3_): *δ* 7.19–7.34 (m, 5H, Ar-H), 6.96 (d, *J =* 8.60 Hz, 2H, Ar-H), 6.65 (dd, *J* = 4.62, 4.62 Hz, 1H, -CH=), 6.59 (d, *J =* 8.10 Hz, 2H, Ar-H), 6.23 (tt, *J =* 5.20, 5.40 Hz, 1H, CH-P), 4.73–4.77 (m, 4H, CH_2_O), 2.22 (s, 3H, CH_3_), 4.38 (dd, *J =* 5.70, 5.70 Hz, 1H, -CH=), 1.33–1.36 (m, 4H, CH_2_), 1.24–1.26 (m, 6H, CH_3_);^ 13^C-NMR (CDCl_3_): *δ* 144.3, 136.4, 133.0, 129.8, 128.6, 127.8, 126.5, 123.8, 68.4, 55.0, 53.8, 24.0, 20.4, 10.0; ^31^P-NMR (CDCl_3_): *δ* 23.0; Anal. Calcd for C_22_H_30_NO_3_P: C 68.20, H 7.80, N 3.62; Found: C 68.01, H 7.85, N 3.53; Analysis: Daicel Chiralpak IA (hexane-EtOH = 95:5 / *v*/*v*), Flow rate = 1.0 mL/min, UV = 254 nm, t_R _(minor) = 7.70 min (*R*), t_R _(major) = 8.40 min (*S*). *e.e.* 15.3%.

*Diisopropyl 1-[N-(4-methylphenyl)amino]-3-phenyl-2-propenylphosphonate* (**9o**): Yellow oil; yield 30%; (KBr cm^-1^): *ν* 3300 (N-H), 2978 (C-H), 1616 (C=C), 1519 (C=C), 1250 (P=O), 989 (P-O-C);^ 1^H-NMR (CDCl_3_): *δ* 7.19–7.34 (m, 5H, Ar-H), 6.96 (d, *J =* 8.00 Hz, 2H, Ar-H), 6.67 (dd, *J* = 5.20, 5.20 Hz, 1H, -CH=), 6.23 (tt, *J =* 5.20, 5.40 Hz, 1H, CH-P), 4.14-4.47 (m, 2H, CH-O), 4.32 (dd, *J* = 6.30, 6.30 Hz, 1H, -CH=), 2.21 (s, 3H, CH_3_), 1.28–1.31 (m, 12H, CH_3_),1.27 (t, *J =* 7.60 Hz, 12H, CH_3_);^ 13^C-NMR (CDCl_3_): *δ* 144.4, 136.5, 132.6, 129.8, 128.5, 127.7, 126.6, 124.2, 113.9, 71.6, 55.5, 54.2, 24.3, 23.9, 20.4; ^31^P-NMR (CDCl_3_): *δ* 21.1; Anal. Calcd for C_22_H_30_NO_3_P: C 68.20, H 7.80, N 3.62; Found: C 68.11, H 7.65, N 3.44; Analysis: Daicel Chiralpak IA (hexane-EtOH = 90:10 / *v*/*v*), Flow rate = 1.0 mL/min, UV = 254 nm, t_R _(minor) = 6.12 min (*R*), t_R _(major) = 6.57 min (*S*). *e.e.* 51.5%.

*Di-n-butyl 1-[N-(4-methylphenyl)amino]-3-phenyl-2-propenylphosphonate* (**9p**): Yellow oil; yield 39%; IR (KBr cm^-1^): *ν* 3444 (N-H), 2958 (C-H), 1683 (C=C), 1521 (C=C), 1234 (P=O), 1022 (P-O-C);^ 1^H-NMR (CDCl_3_): *δ* 7.19–7.34 (m, 5H, Ar-H), 6.96 (d, *J =* 8.00 Hz, 2H, Ar-H), 6.67 (dd, *J =* 3.50, 6.30 Hz, 1H, -CH=), 6.60 (d, *J =* 8.00 Hz, 2H, Ar-H), 6.23 (tt, *J =* 5.20, 5.70 Hz, 1H, CH-P), 4.41 (dd, *J =* 6.30, 6.30 Hz, 1H, -CH=), 4.05–4.15 (m, 8H, 4CH_2_O), 2.22 (s, 3H, CH_3_), 1.56–1.64 (m, 4H, CH_2_), 1.33–1.39 (m, 4H, CH_2_), 0.86–0.90 (m, 6H, CH_3_);^ 13^C-NMR (CDCl_3_): *δ* 144.2, 136.4, 132.9, 129.8, 128.5, 127.8, 126.6, 123.8, 114.0, 66.7, 55.0, 53.8, 32.6, 20.4, 18.7, 13.6; ^31^P-NMR (CDCl_3_): *δ* 22.9; Anal. Calcd for C_24_H_34_NO_3_P: C 69.37, H 8.25, N 3.37; Found: C 69.26, H 8.09, N 3.22; Analysis: Daicel Chiralpak IA (hexane-EtOH = 90:10 / *v*/*v*), Flow rate = 1.0 mL/min, UV = 254 nm, t_R _(minor) = 6.55 min (*R*), t_R _(major) = 7.05 min (*S*). *e.e.* 22.7%.

## 4. Conclusions

In summary, we have employed and studied the role of axially chiral binaphthyl phosphoric acid **6a** in the asymmetric hydrophosphonylation of different aldimines with dialkyl phosphites. The desired *α*-aminophosphonates **9a-9p **could be obtained in moderate yields and enantioselectivity. Although the presence of bulky substituents at the 3, 3’ positions of the catalyst appears to influence the outcome of the reaction, the enantioselectivity is still largely governed by the combined nature of the substrate and the catalyst structure. Further application of similar organocatalysts obtained by making subtle structural variation in the parent moiety is currently underway. 
